# Meloxicam Executes Its Antitumor Effects against Hepatocellular Carcinoma in COX-2- Dependent and -Independent Pathways

**DOI:** 10.1371/journal.pone.0092864

**Published:** 2014-03-27

**Authors:** Xiaofeng Dong, Rui Li, Peng Xiu, Xuesong Dong, Zongzhen Xu, Bo Zhai, Feng Liu, Hongchi Jiang, Xueying Sun, Jie Li, Haiquan Qiao

**Affiliations:** 1 Department of General Surgery, Qianfoshan Hospital, Shandong University, Jinan, China; 2 Department of General Surgery, Liaocheng People's Hospital, Liaocheng, China; 3 The Hepatosplenic Surgery Center, Department of General Surgery, The First Affiliated Hospital of Harbin Medical University, Harbin, China; 4 Department of Molecular Medicine and Pathology, Faculty of Medical and Health Sciences, University of Auckland, Auckland, New Zealand; Institut für Pathologie, Greifswald, Germany, Germany

## Abstract

**Background:**

Cyclooxygenase (COX)-2 is overexpressed in many types of cancers including hepatocellular carcinoma (HCC). Meloxicam, a selective COX-2 inhibitor, has shown potential therapeutic effects against HCC, but the mechanisms accounting for its anti-cancer activities remain unclear.

**Methods and Findings:**

Meloxicam inhibited the ability of human HCC cells expressing higher levels of COX-2 to migrate, invade, adhere and form colonies through upregulating the expression of E-cadherin and downregulating the expression of matrix metalloproteinase (MMP) -2. Meloxicam induced cell apoptosis by upregulating pro-apoptotic proteins including Bax and Fas-L, and downregulating anti-apoptotic proteins including survivin and myeloid cell leukemia-1 (Mcl-1), through inhibiting phosphorylation of AKT. Addition of prostaglandin E2 (PGE2), the major product of COX-2, could abrogate the effects of meloxicam on the expression of survivin and myeloid cell leukemia-1 (Mcl-1), but not Bax and Fas-L, indicating that meloxicam induces cell apoptosis via both COX-2-dependent and -independent pathways. Meloxicam also induced cell autophagy by upregulating Beclin 1 and light chain 3-II. Specific inhibition of autophagy by 3-methyladenine and chloroquine had little effect on cell apoptosis but could enhance the pro-apoptotic effects of meloxicam by further upregulating the expression of Bax.

**Conclusions:**

Meloxicam executes its antitumor effects by targeting the COX-2/MMP-2/E-cadherin, AKT, apoptotic and autophagic pathways in COX-2-dependent and -independent pathways, and inhibition of cell autophagy could help to overcome the resistance to meloxicam-induced apoptosis in HCC.

## Introduction

Hepatocellular carcinoma (HCC) is the second most frequent cause of cancer death in men worldwide [1], and is extremely resistant to the chemotherapeutic drugs [2]. Sorafenib is the unique first-line drug recommended for advanced HCC, but it has not been widely accepted as it only prolongs 2–3 month of survival of advanced HCC patients compared to placebo [2], and it is cost-prohibitive in Asia and sub-Saharan Africa, which have the highest incidence of HCC [1]. Therefore, it is urgently required to seek novel drugs to combat HCC.

Cyclooxygenase (COX)-2, a rate-limiting enzyme in the synthesis of prostaglandin (PG), has emerged as an anti-cancer target, as it is overexpressed in many types of cancers including HCC [3]. COX-2 influences all aspects of cancer, such as cell proliferation, motility, survival, invasiveness and apoptosis resistance [4]–[7]. We have previously reported that meloxicam, a selective COX-2 inhibitor, suppressed the growth and induced apoptosis of HCC cells [8], [9]. However, the mechanisms particularly the molecular signaling pathways involved in its anti-cancer effects remain unclear.

PGE2 is the predominant product of COX-2, and is the most abundant among the PGs produced by COX-2-overexpressing tumors [7]. PGE2 activates diverse biological effects primarily through its binding to a family of receptors, such as prostaglandin E2 receptor (EP2) [10], [11]. The biological relevance of EP2-dependent signaling to the oncogenic effects of PGE2 is corroborated by the impairment of tumor cell growth, invasion, and metastatic dissemination in EP2-knockout animals [12]. PGE2 induce activation of AKT, which could be blocked by antagonists of PGE2, in prostate cancer cells [13]. AKT is activated in a high proportion of HCC tissues [14], and the AKT signaling pathway is critical for cancer development and progression by regulating downstream targets involved in apoptosis of cancer cells [15]. These studies indicate that inhibition of COX-2 activity by meloxicam may execute its antitumor effects by blocking PGE2-regulated cell signaling pathways.

Autophagy was initially referred as a self-digestion process [16], later was found to induce cell death and considered to be Type II programmed cell death (PCD) [17]. Now it is accepted that autophagy acts as a double-edged sword for cancer cells depending on the cellular context and the stimuli [18]. The autophagic pathway crosstalks with apoptosis, and the mutiple molecular nodes of crosstalks present many opportunities for therapeutic intervention [18], making it a hot spot for cancer research. However, the role Autophagy in meloxicam-mediated apoptosis of cancer remains unknown.

Therefore, we designed the present study to investigate the underlying mechanisms of meloxicam in HCC cancer cells. Here we have provided evidence that meloxicam executes its antitumor effects against HCC by regulating the COX-2/ matrix metalloproteinase (MMP)-2/E-cadherin, AKT, apoptotic and autophagic pathways via both COX-2-dependent and -independent ways.

## Materials and Methods

### Cell culture

Human HCC cell lines, HepG2, Bel-7402 and Huh-7, were obtained from the American Type Culture Collection, Rockville, Maryland, USA, and SMMC-7721 and SMMC-7402 from the Type Culture Collection Cell Bank, Chinese Academy of Science, Shanghai, China. The cells were routinely cultured in Dulbecco's modified Eagle's medium (DMEM) supplemented with 10% fetal bovine serum, 100 U/mL penicillin, and 100 μg/mL streptomycin at 37°C in 95% air and 5% CO_2_.

### Cell viability assay

The Cell Counting Kit-8 (CCK-8) (Dojindo Molecular Technologies, Inc., Beijing, China) was used to determine cell viability. Briefly, cells (5×10^3^/well) were incubated with meloxicam-containing DMEM in 96-well plates for 72 h, and then the culture medium was replaced with fresh medium containing 10 μl of CCK-8 solution. The cells were further incubated for 2 h at 37°C, and the optical density (OD) at 450 nm was measured. The viability inhibitory rate was calculated as: (control OD value - experiment OD value)/control group OD value × 100%.

### Antibodies and reagents

Antibodies (Abs) against MMP-1, MMP-2, COX-2, myeloid cell leukemia-1 (Mcl-1), Bax, Fas, Fas-L, AKT, phosphorylated AKT (p-AKT) (Ser473), survivin, Beclin-1 and light chain 3 (LC3) were purchased from Cell Signaling Technology (Danvers, Massachusetts, USA). Abs against E-cadherin and EP2 were from Abcam (Hong Kong, China). PGE2 protein, recombinant human MMP-2 protein (rh-MMP-2) and meloxicam dissolved in dimethyl sulfoxide (DMSO) were purchased from Merck Millipore (Merck Millipore, Darmstadt, Germany). Matrigel basement matrix (10 mg/ml) was purchased from BD Biosciences (San Jose, CA, USA), MK-2206 (an AKT inhibitor) from Jinan Trio Pharmatech Co., Ltd. (Jinan, China), and two autophagy inhibitors 3-methyladenine (3-MA) and chloroquine (CQ) from Sigma-Aldrich (Shanghai, China).

### Cell migration and invasion assays

The methods have been described previously [19], [20]. Briefly, 1×10^5^ cells, in 300 μl of DMEM (with 1% FBS) containing meloxicam (80 μM) were seeded to the upper chamber of Transwells (Corning, New York, USA). The bottom wells of the chambers were filled with 500 μl DMEM (with 10% FBS). After 48 h incubation, transwells were fixed with 95% ethanol and then stained with 1% crystal violet. Images of three different fields (×100 magnification) were captured from each membrane, and the number of migrated cells counted. Similarly, the cell invasion assay was performed by adding Matrigel Basement Matrix to the upper chamber of transwells.

### Cell adhesion assay

The methods have been described previously [19], [20]. The 24-well plates were coated with collagen I (5 μg/cm^2^). The cells which had been incubated with meloxicam (80 μM) for 48 h were seeded at a density of 1×10^5^/well, and were further incubated for 80 min. The non-adherent cells were washed away, and remaining cells were counted under a microscope in six randomly-selected fields (×100 magnification).

### Colony formation Assay

The methods have been described previously [19], [20]. Cells (1×10^3^) were seeded in the plastic 5-cm dishes, and meloxicam (80 μM) was added 24 h later. The cells were further incubated for 3 weeks. The cells were fixed with 95% ethanol and stained with 0.1% crystal violet, and photographed. The colonies containing more than 50 cells were counted.

### Reverse-Transcription Polymerase Chain Reaction (RT-PCR)

Total RNA was extracted from the cells using Trizol reagent (Invitrogen), and cDNA was synthesized by using a cDNA synthesis kit (Invitrogen). The reaction mixtures for quantitative RT-PCR were prepared (Shanghai Sunbio Medical Biotechnology, Shanghai, China) with the primers targeting MMP-2 (5′-TGACGGTAAGGACGGACTC-3′; 5′-ATACTTCACACGGACCACTTG-3′), E-cadherin (5′-TGCCCAGAAAATGAAAAAGG-3′; 5′-GGATGACAGCGTGAGAGA-3′) and GAPDH (5′-TTACTCCTTGGAGGCCATGTGGGC-3′; 5′-ACTGCCACCCAGAAGACTGTGGATGG-3′), and analyzed by MX3000P Real-time PCR systems (Stratagene, Wilmington, DE, USA). Experiments were performed in triplicate, and the data were calculated by ΔΔCt methods. In addition, a standard PCR was also carried out to generate products of 124 bp, 223 bp and 465 bp, for MMP-2, E-cadherin and GAPDH, respectively. The PCR products were resolved on 2% agarose gels, and visualized under ultraviolet light.

### Enzyme-linked immunosorbent assay (ELISA)

The concentrations of sE-cadherin (soluble E-cadherin) in supernatants of cell culture were measured using a commercial Human sE-cadherin quantikine ELISA Kit (R&D Systems, Inc., Minneapolis, MN, USA). Absorbance at 450 nm was read and corrected by using the 540 nm reading on a microtiter plate reader (Powerwave X Bio-tek Instruments Inc, USA). Concentrations of sE-cadherin were determined against a standard curve.

### Apoptosis assay

The cells were incubated with 5 μl of Annexin V and 5 μl of propidium iodide (PI) for 15 min at room temperature in dark, according to the manufacturer's instruction (BD Biosciences, San Jose, CA), and then subjected to flow cytometry to measure the apoptosis rate (%).

### Staining for cell autophagy

The cells were first incubated with acridine orange (5 μM) (Sigma-Aldrich) at 37°C for 15 min, washed with cold PBS, and then examined under the inverted fluorescent microscopy. Autophagic lysosomes appeared as orange/red fluorescent cytoplasmic vesicles, while nuclei were stained green. Alternatively, acridine orange-stained cells were trypsinized, washed, and analyzed on a FACScalibur flow cytometer (BD. Biosciences, San Jose, California, USA). The degree of autophagic lysosome was expressed as fold change of acridine orange fluorescence intensity (FL3) of red in treated cells versus control cells. The cells were also stained by monodansylcadaverine (MDC) (Sigma-Aldrich) for detecting autophagic autophagosomes. Briefly, cells were incubated with 0.05 mM MDC (Sigma-Aldrich) at 37°C for 15 min, washed with cold PBS, and examined under microscopy. To quantify the autophagosomes, the cells were suspended with 0.05 mM MDC at 37°C for 30 min and then subjected to flow cytometry (BD. Biosciences, San Jose, California, USA) to measure the MDC-positive cells.

### Western blot analysis

Protein concentrations in cell extracts were determined (Bio-Rad, Richmond, California, USA). Briefly, equal amounts of protein fractions of lysates were resolved over SDS-PAGE gels, transferred to PVDF membrane, and immunoblotted as previously described [19]–[23]. Protein band intensities were quantified by densitometric analysis using ImageJ software (National Institutes of Health, USA).

### Statistical analysis

The data are expressed as mean ± standard deviation (SD). Comparisons were made using a one-way ANOVA followed by Dunnett's test with SPSS software (version 17.0, SPSS China, Shanghai, China). *P*<0.05 was considered statistically significant.

## Results

### HCC cells express COX-2 and EP2 proteins and responds to meloxicam treatments

**Figure 1:**
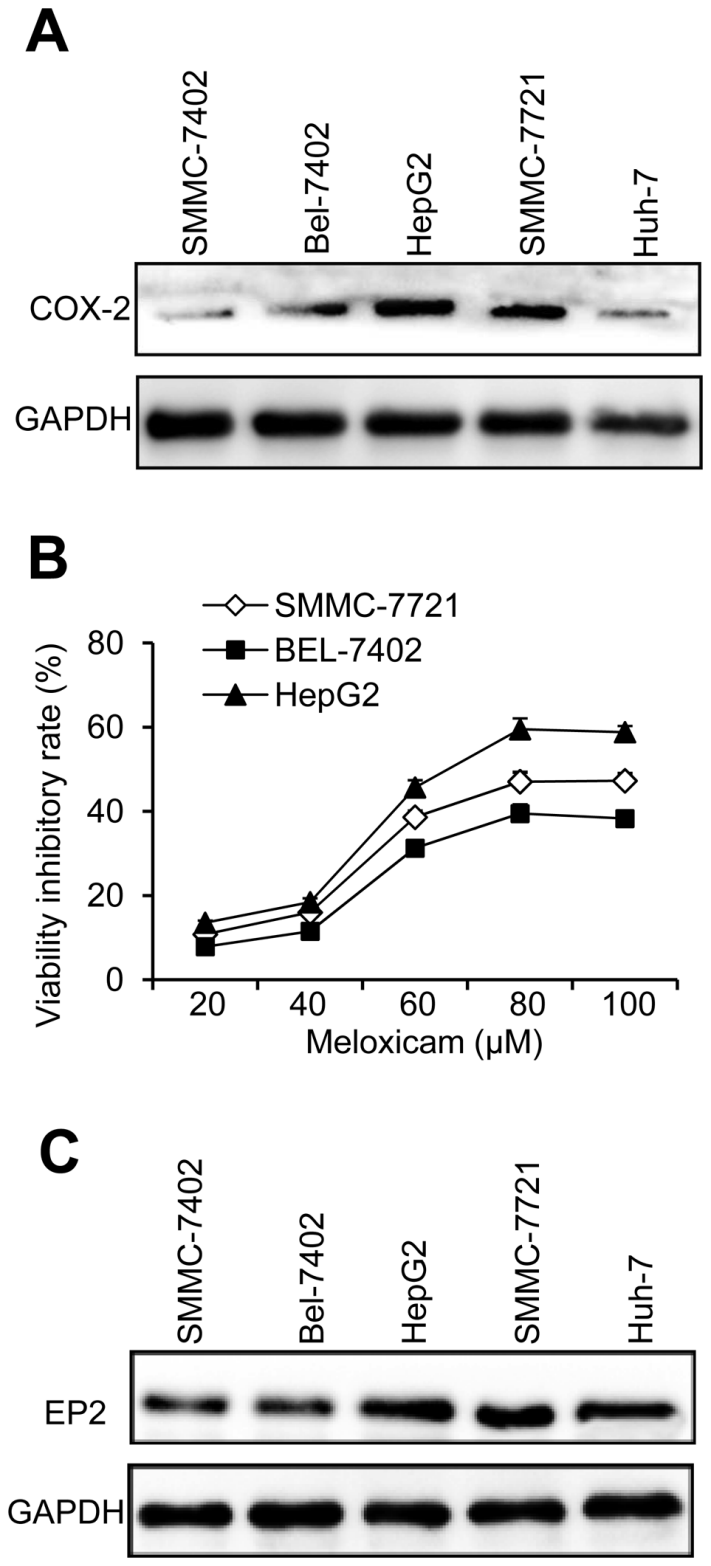
The expression of COX-2 and EP2 in HCC cells and meloxicam reduces cell viability *in vitro*. (A) The expression of COX-2 in HCC cell lines SMMC-7402, Bel-7402, HepG2, SMMC-7721 and Huh-7 was detected by Western Blotting. GAPDH served as an internal control. (B) Bel-7402, HepG2 and SMMC-7721 cells that express higher levels of COX-2 were incubated with increasing concentrations of meloxicam, and the rates of viability inhibition were measured. (C) The expression of EP2 in the above five HCC cell lines was detected by Western Blotting. GAPDH served as an internal control.

We first examined the levels of COX-2 protein expressed by the available human HCC cell lines by Western blot analysis. The cell lines expressed different levels of COX-2 protein, thus Bel-7402, HepG2 and SMMC-7721 cells intensely expressed COX-2, whereas SMMC-7402 and Huh-7 cells showed weak expression of COX-2 (Fig. 1A). Based on the data, three cell lines, Bel-7402, HepG2 and SMMC-7721, were selected for testing the inhibitory effects of meloxicam on the viability of cells. The cells were incubated with culture media containing 20, 40, 60, 80 or 100 μM of meloxicam for 72 h, and the viability inhibitory rates were measured. Meloxicam reduced the viability of the three cell lines in a concentration-dependent manner, and meloxicam at a concentration of 80 μM induced the highest inhibitory rates for the three cell lines and this concentration was used for the following experiments. (Fig. 1B). We further exmined the expression of EP2, and the results showed that all the five HCC cell lines expressed EP2 protein at different levels (Fig. 1C).

### Meloxicam inhibits migration, invasion, adhesion and colony formation of HCC cells

**Figure 2:**
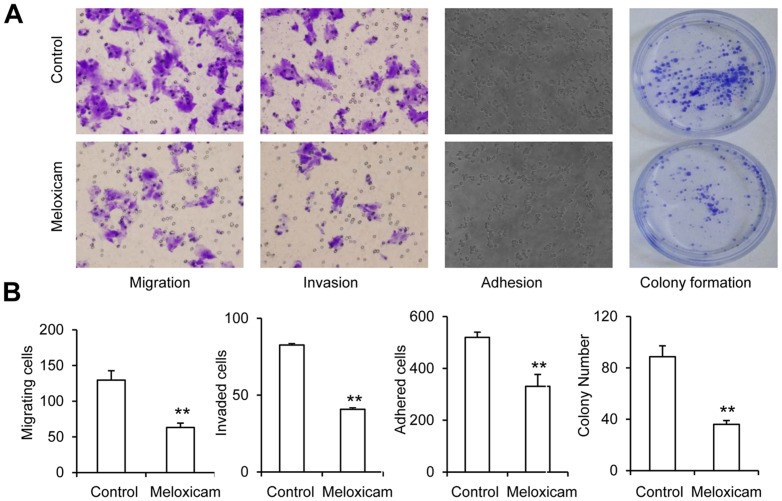
Meloxicam inhibits cell migration, invasion, adhesion and colony formation. (A) Representative photographs were taken from HepG2 cells incubated for 48 h with meloxicam (80 μM) or vehicle (control) and subjected to cell migration, invasion, adhesion and colony formation assays as described in Materials and Methods. (B) The above assays were quantified. Data represent three independent experiments. “**” indicates a highly significant (*P*<0.001) difference from controls.

HepG2 cells were incubated with meloxicam (80 μM) or vehicle (control) and subjected to cell migration, invasion, adhesion and colony formation assays. As shown in the representative images (Fig. 2A), the cells pre-incubated with meloxicam had fewer cells that migrated, invaded and adhered, and fewer colonies formed, than untreated controls. The data were further quantified, and as shown in Fig. 2B, meloxicam significantly (All *P*<0.001) reduced the ability of HepG2 cells to migrate, invade and adhere, and to form colonies, compared with untreated controls. The results were further confirmed by using another HCC cell line, SMMC-7721 (Fig. S1).

### Meloxicam regulates the expression of MMP-2 and E-cadherin in a COX-2- dependent way

**Figure 3:**
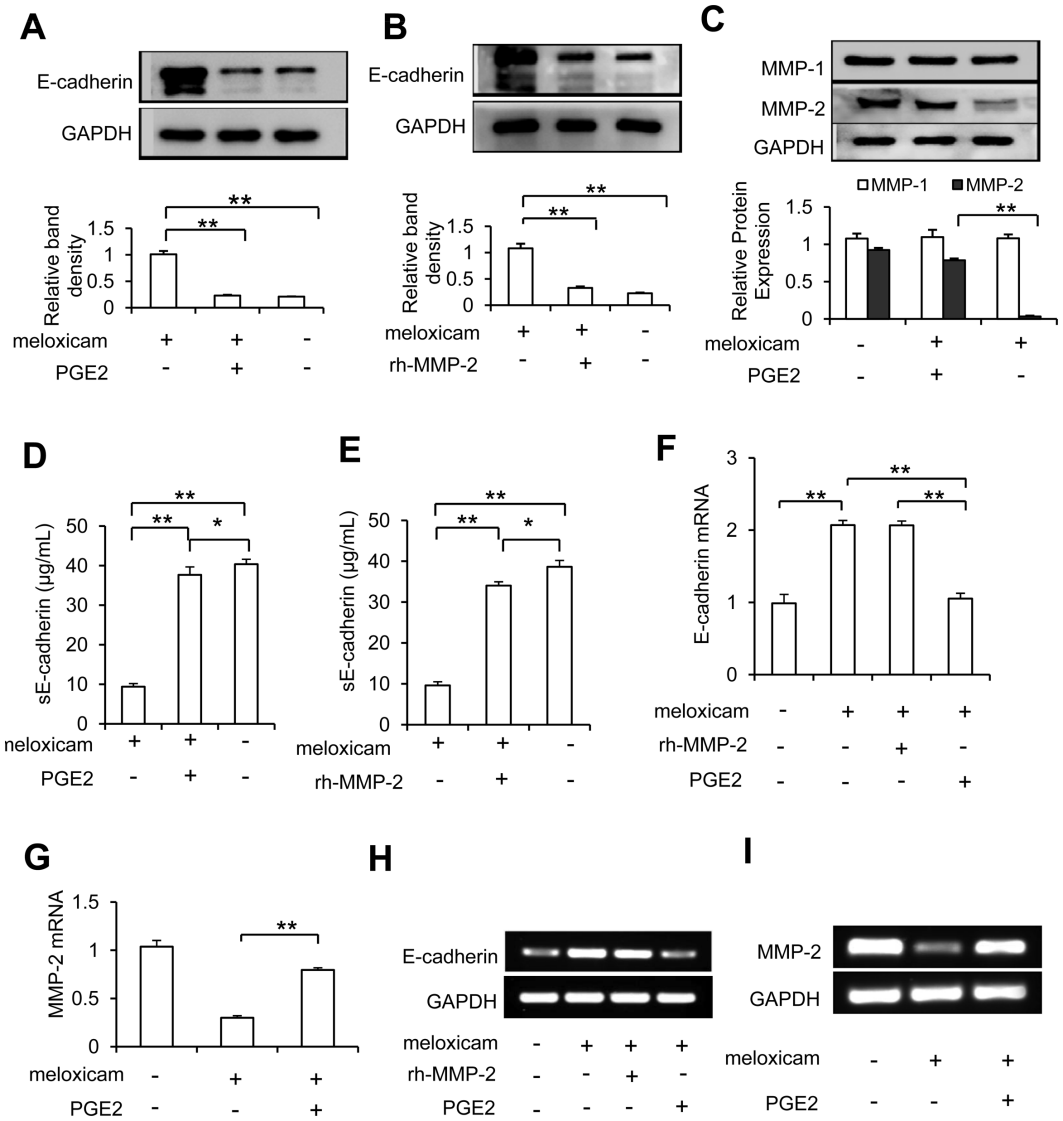
Meloxicam upregulates E-cadherin and downregulates MMP-2 in a COX-2-dependent way. HepG2 cells were cultured for 72(80 μM), PGE2 (3 μM) or rh-MMP-2 (25 ng/mL), or the combination. Cell lysates were analyzed by Western blot analysis to detect expression of E-cadherin (A, B) and MMP-1/MMP-2 (C) proteins. The band density in each assay was measured and normalized to that of GAPDH, respectively. (D, E) The concentrations of soluble E-cadherin (sE-cad) in supernatants from the above cell culture were measured by ELISA. (F–H) The above cells were lysed and subjected to quantitative real-time RT-PCR for measuring the levels of E-Cadherin (F) and MMP-2 (G) mRNAs, and to a standard RT-PCR assay, in which PCR products of E-Cadherin (I) and MMP-2 (H) were electrophoresed. GAPDH served as an internal control. Data represent three independent experiments. “*” indicates a significant (*P*<0.05) difference, and “**”, a highly significant (*P*<0.001) difference.

Meloxicam treatment significantly upregulated the expression of E-cadherin, but this effect of meloxicam could be almost abrogated by addition of PGE2 in HepG2 cells (Fig. 3A). Addition of rh-MMP-2 could also neutralized the upregulation of E-cadherin by meloxicam (Fig. 3B), indicating that meloxicam may regulate the expression of E-cadherin through its effect on MMP-2. Meloxicam significantly reduced the expression of MMP-2 but not MMP-1 proteins, and addition of PGE2 could abrogate this effect (Fig. 3C). Meloxicam treatment significantly reduced the sE-cadherin levels in the supernatants of cell culture, and both PGE2 and rh-MMP-2 neutralized this reduction (Fig. 3D and 3E). We next examined the expression of E-cadherin and MMP-2 mRNAs. Meloxicam treatment resulted in increased expression of E-cadherin mRNA that could be abrogated by addition of PGE2 but not rh-MMP-2 (Fig. 3F), and reduced expression of MMP-2 mRNA that could not be abrogated by PGE2 (Fig. 3G), as measured by qRT-PCR. A standard RT-PCR was also performed to show the consistency of the qRT-PCR assays (Fig. 3H and 3I).

### Meloxicam regulates phosphorylation of AKT in a COX-2-dependent way

**Figure 4:**
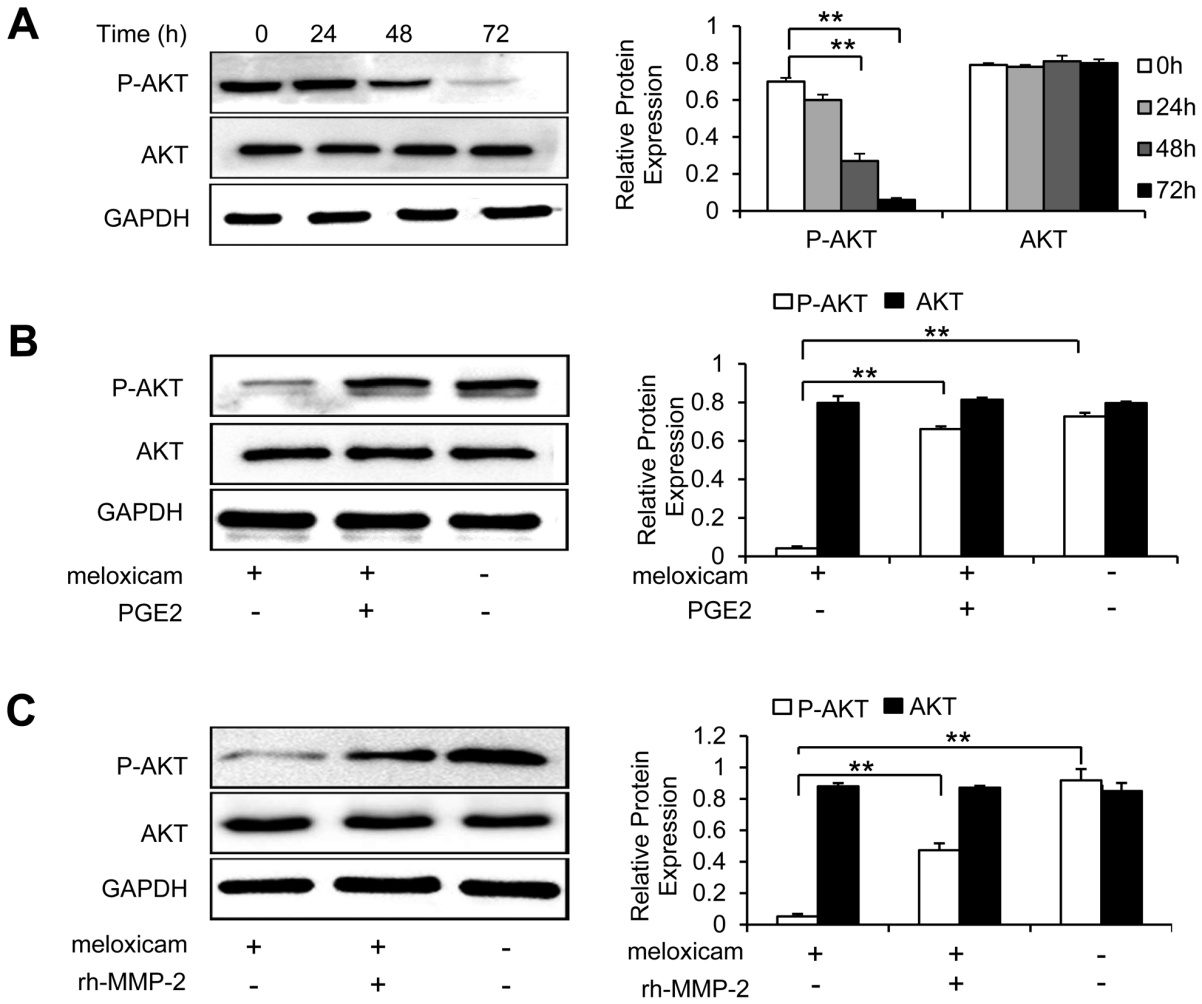
Meloxicam inhibits phosphorylation of AKT in a COX-2-depdendent way. (A) HepG2 cells were incubated with meloxicam (80 μM), and harvested at the indicated time points. (B, C) HepG2 cells were incubated for 72 h with meloxicam (80 μM) in the presence and absence of PGE2 (3 μM) (B) or rh-MMP-2 (25 ng/mL) (C). The above harvested cells were subjected to Western blot analysis. The band density in each assay was measured and normalized to that of GAPDH, respectively. Data represent three independent experiments. “**”, indicates a highly significant (*P*<0.001) difference.

The AKT pathway plays a critical role in regulating cell growth, proliferation, survival and motility, which drive tumor progression [15]. Therefore, we examined whether meloxicam has effects on the activation of AKT. As shown in Fig. 4A, incubation of HepG2 cells with meloxicam (80 μM) down-regulated the expression of P-AKT, an active form of AKT, in a time-dependent manner, while the expression of total AKT remained unchanged. However, both PGE2 and rh-MMP-2 abrogated the inhibitory effects of meloxicam on the activation of AKT (Fig. 4 B and 4C).

### Meloxicam induces cell apoptosis in COX-2-dependent and -independent pathways

**Figure 5:**
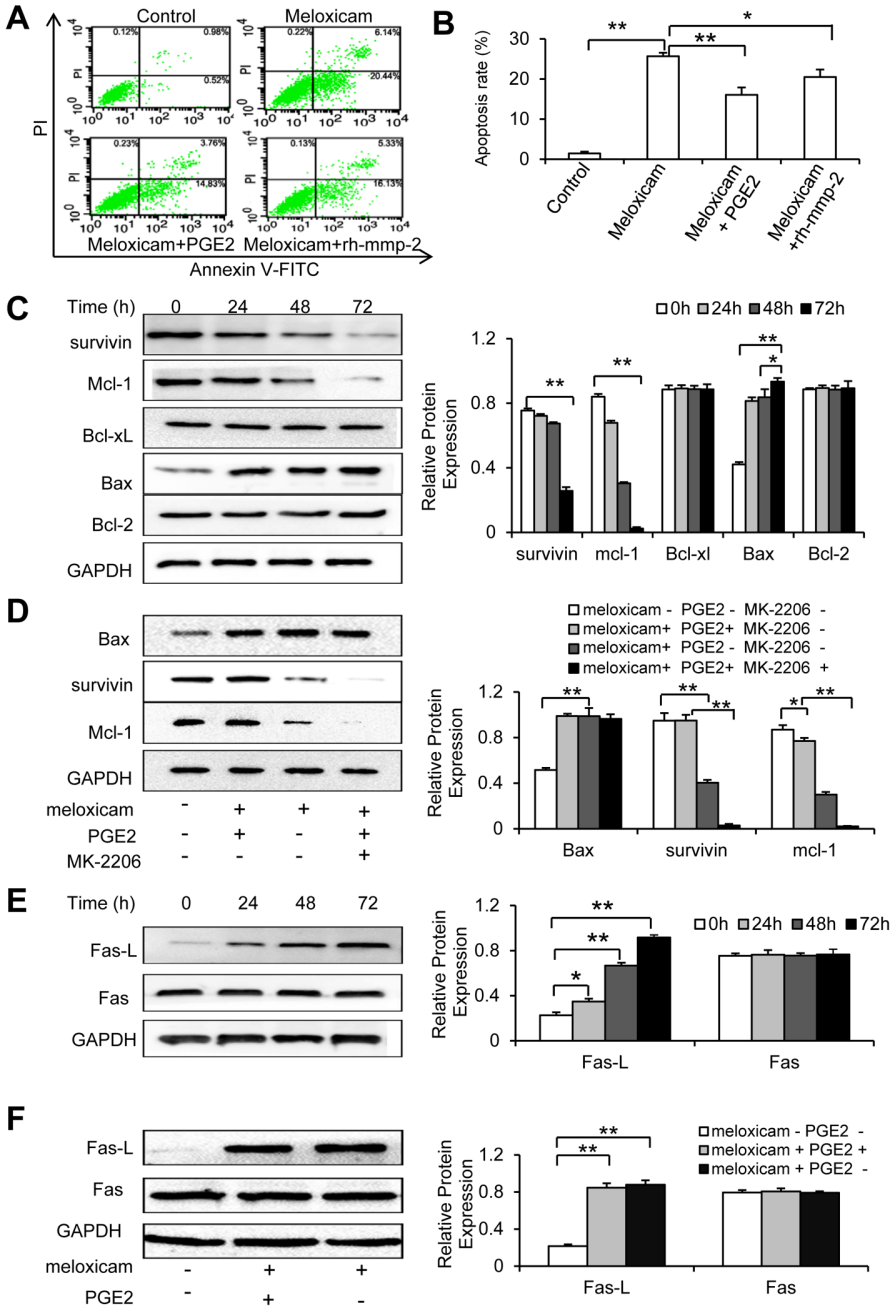
Meloxicam induces cell apoptosis via COX-2-dependent and -independent mechanisms. (A, B) HepG2 cells were incubated for 72 h with meloxicam (80 μM) in the presence or absence of PGE2 (3 μM) or rh-MMP-2 (25 ng/mL). Untreated cells served as controls. (A) Representative dot plots were taken from cytometrically analyzed cells. (B) The apoptosis rate was calculated. (C) HepG2 cells were incubated with meloxicam (80 μM), and harvested at the indicated time points. (D) HepG2 cells were incubated for 72 h with meloxicam (80 μM) in the presence or absence of PGE2 (3 μM) or MK-2206 (5 μM). (E) HepG2 cells were incubated with meloxicam (80 μM), and harvested at the indicated time points. (F) HepG2 cells were incubated with meloxicam (80 μM) in the presence or absence of PGE2 (3 μM) for 72 h, and harvested. The above harvested cells were subjected to Western blot analysis. The band density in each assay was measured and normalized to that of GAPDH, respectively. Data represent three independent experiments. “*” indicates a significant (*P*<0.05) difference, and “**”, a highly significant (*P*<0.001) difference.

We have previously reported that meloxicam induced apoptosis of HepG2 cells [8], [9], which were confirmed by the present results that meloxicam significantly increased the apoptosis rate compared with untreated controls (25.68±0.87% vs. 1.45±0.44%) as measured by flow cytometry (Fig. 5A and 5B). Addition of PGE2 or rh-MMP-2 partially neutralized the pro-apoptotic effects of meloxicam (16.06±1.83% vs. 25.68±0.87%, and 20.51±1.86% vs. 25.68±0.87%, respectively) (Fig. 5 A and 5B). The results were further confirmed by using SMMC-7721 (Fig. S2).

We next examined the expression of apoptosis-related proteins. Incubation of HepG2 cells with meloxicam downregulated the expression of survivin and Mcl-1, and upregulated the expression of Bax, in a time-dependent manner; but had no effect on the expression of Bcl-xL and Bcl-2 (Fig. 5C). Addition of PGE2 abrogated the effects of meloxicam on the expression of Mcl-1 and survivin, but had no effect on the expression of Bax (Fig. 5D). Addition of MK-2206, an AKT specific inhibitor, resulted in almost disappearance of Mcl-1 and survivin proteins in the cells incubated with meloxicam and PGE2, but had no effect on the expression of Bax (Fig. 5D), indicating that meloxicam increased Bax expression in a COX-2-independent way. We further examined the expression of Fas-L and Fas, the key regulators in the extrinsic pathway for apoptosis. Meloxicam upregulated the expression of Fas-L in a time-dependent manner, but had no effect on Fas expression (Fig. 5E). The upregulation of Fas-L by meloxicam could not be neutralized by PGE2, indicating that meloxicam increased Fas-L expression in a COX-2-independent way (Fig. 5F).

### Meloxicam induces autophagy and inhibition of autophagy enhances the pro-apoptotic activity of meloxicam

**Figure 6:**
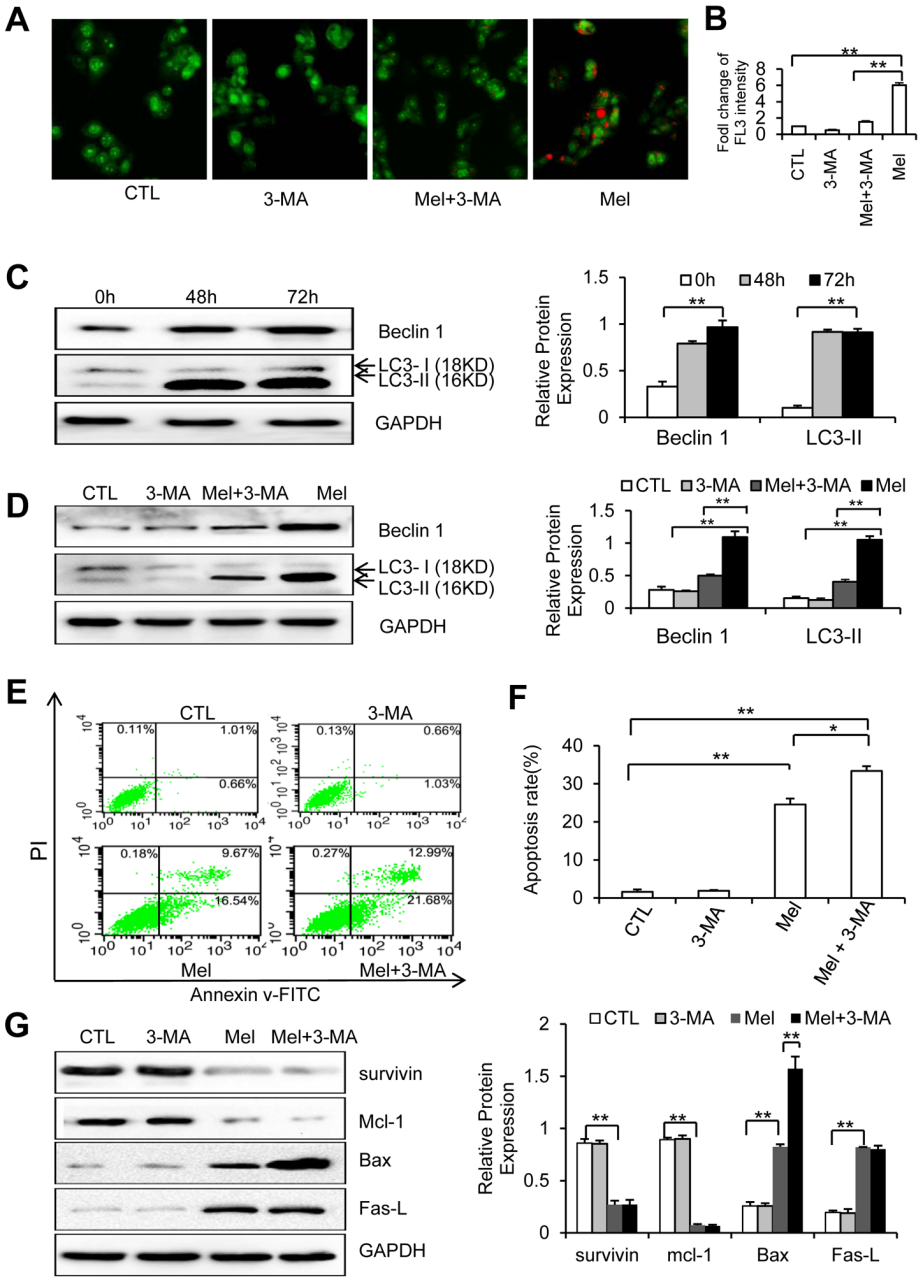
Meloxicam induces autophagy and inhibition of autophagy promotes the apoptosis of HepG2 cells. (A) Representative images were from HepG2 cells that were incubated for 72 h with meloxicam (Mel) (80 μM) in the presence or absence of 3-MA (2 mM), and then stained by acridine orange. Untreated cells served as control (CTL). (B) The above cells from (A) were further subjected to flow cytometry to measure the degree of autophagic lysosomes as expressed by fold change of acridine orange fluorescence intensity (FL3) in treated cells versus control cells. (C) HepG2 cells were incubated with meloxicam (80 μM), and harvested at the indicated time points. (D–F) HepG2 cells were incubated for 72 h with meloxicam (80 μM) in the presence or absence of 3-MA (2 mM). (E) Representative dot plots were taken from cytometrically analyzed cells. (F) The apoptosis rate in (E) was calculated. (G) The above harvested cells were subjected to Western blot analysis. The band density in each assay was measured and normalized to that of GAPDH, respectively. Data represent three independent experiments. “*” indicates a significant (*P*<0.05) difference, and “**”, a highly significant (*P*<0.001) difference.

Meloxicam induced autophagy of HepG2 cells evidenced by meloxicam-treated cells had more acidic vesicles stained orange-red fluorescence in acridine orange staining assay (Fig. 6A). Quantitative analysis of autophagy by flow cytometry showed that meloxicam significantly increased cell autophagy evidenced the increased FL3 intensity compared to untreated cells, and 3-MA abrogated this increase (Fig. 6B). The similar results were obtained by using another HCC cell line, SMMC-7721 (Fig. S3). Cell autophgy was also detected by staining the cells with MDC, a specfic autophagosome marker, and similar results were obtained in both HepG2 and SMMC-7721 cells (Fig. S4). The results were further confirmed by the upregulation of Beclin 1 and LC3-II, the two autophagy markers, induced by meloxicam, in a time-dependent manner (Fig. 6 C). However, 3-MA significantly reduced meloxicam-induced autophagy and upregulation of Beclin 1 and LC3-II (Fig. 6A and 6D). The functional relationship between apoptosis and autophagy is complex in a sense that autophagy suppresses apoptosis under certain circumstances, whereas it constitutes an alternative cell-death pathway in other cellular settings [24]. Here, we showed that inhibition of autophagy by 3-MA had little effect on apoptosis, but synergized with meloxicam in inducing apoptosis of HepG2 cells (Fig. 6 E and 6F). The similar results were obtained by using another HCC cell line, SMMC-7721 (Fig. S5). The results were further confirmed by applying chloroquine, another autophagy inhibitor, in both HepG2 and SMMC-7721 cells (Fig. S6). We next examined the expression of apoptosis-associated proteins including survivin, Mcl-1, Bax and Fas-L, which had been shown to be regulated by meloxicam (Fig. 5). As shown in Fig. 6G, 3-MA itself had little effect on the expression of the above proteins, but could synergize with meloxicam to further increase the expression of Bax, but not the other three proteins.

## Discussion

**Figure 7:**
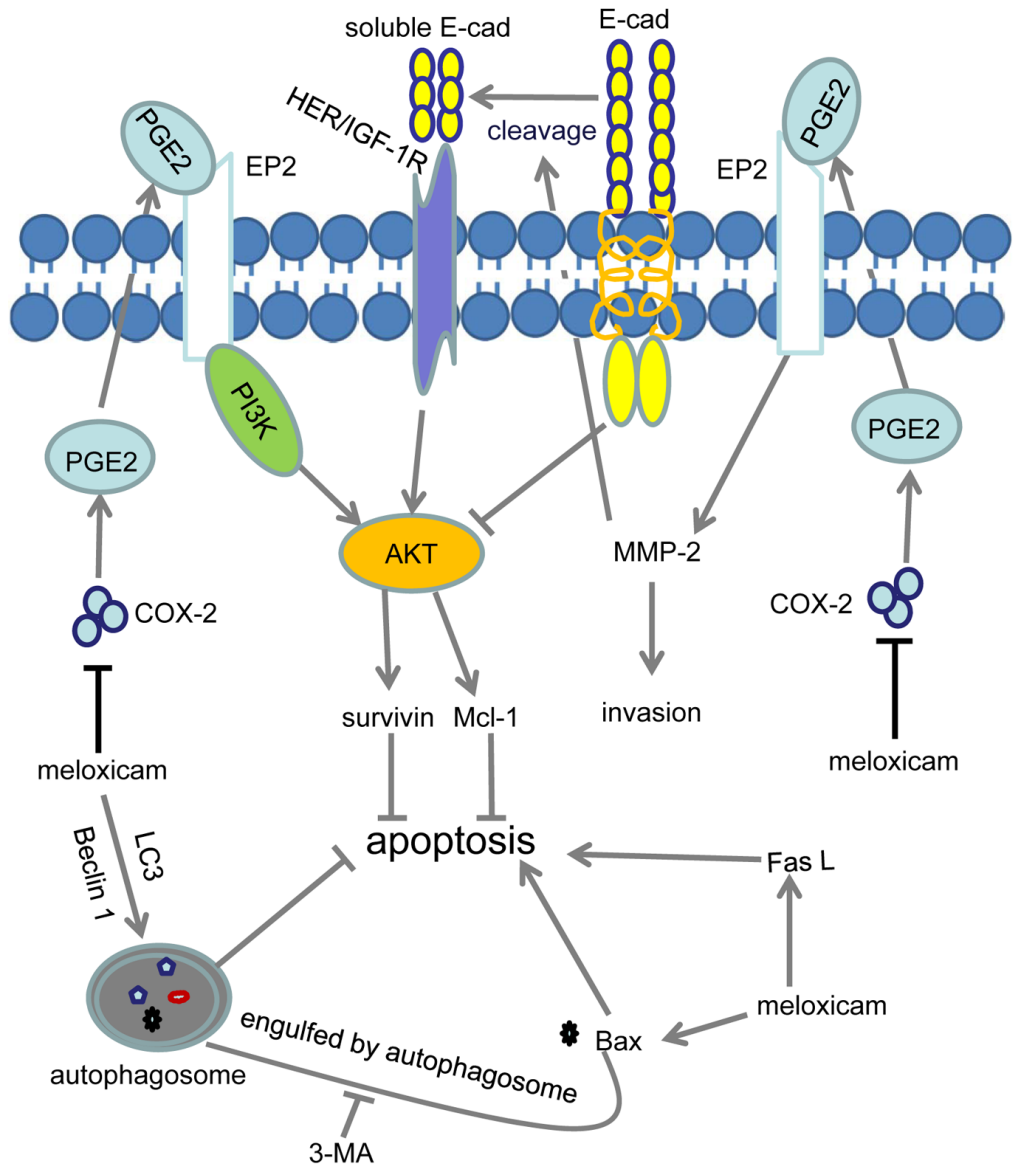
Proposed mechanisms by which meloxicam executes its antitumor effects in COX-2-depdendent and -independent ways. Meloxicam inhibits the production of PGE2 by inhibiting COX-2 activity. PGE2 binds to EP2 to upregulate the expression of survivin and Mcl-1 via activation of AKT. Meloxicam induces cell apoptosis by upregulating Bax and Fas-L in a COX-2-independent way. 3-MA inhibits the engulfing of Bax by autophagosome, thus blocks the inhibitory effect of autophagy on apoptosis. PGE2 binds to EP2 to upregulate MMP-2, which in turn promotes the cleavage of E-cadherin. Soluble E-cadherin binds to HER/IGF-1R to activate AKT, while full-length of E-cadherin inhibits activation of AKT. “→” indicates positive regulation or activation; “⊥”, negative regulation or blockade; COX-2, cyclooxygenase-2; E-cad, E-cadherin; EP2, prostaglandin E2 receptor; HER, human epidermal growth factor receptor; IGF-1R, insulin-like growth factor-1 receptor; Mcl-1, myeloid cell leukemia-1; MMP-2, matrix metalloproteinase-2; PGE2, prostaglandin E2; 3-MA, 3-methyladenine.

The present study has demonstrated that meloxicam executes its antitumor effects against HCC by inhibiting cell migration, invasion, adhesion and colony formation, and inducing cell autophagy and apoptosis, in both COX-2-depedent and -independent pathways. COX-2 is constitutively overexpressed in many human premalignant, malignant and metastatic epithelial tumors including HCC [25]. Upregulated expression of COX-2 is an early event during carcinogenesis, and is associated with poor prognosis as it promotes tumor cell proliferation, invasion and metastasis [7]. COX-2 elicits its effects mainly through producing prostaglandins, such as PGE2, on cancer cells [7], as PGE2 activates cellular signaling pathways through its binding to EP2 [10], [11] (Fig. 7). The results presented herein indicate that meloxicam executes its antitumor activities through the COX-2/MMP-2/E-cadherin, AKT, apoptotic and autophagic pathways in HCC.

It has been reported that the COX-2/E-cadherin pathway is involved in the metastasis and invasion of HCC cells [26], and MMPs promote tumor metastasis and invasion by degrading extracellular matrix proteins [27]. E-cadherin is considered as a suppressor for cancer invasion and metastasis [28], [29]. Lower expression of E-cadherin is positively associated with more malignant behavior and higher potential of invasion and metastasis in HCC patients [29]–[31]. Elevated tumoral COX-2 expression is associated with tumor invasion, metastasis, and poor prognosis, and the COX-2-dependent pathways contribute to the modulation of E-cadherin expression [32], [33]. Our results showed that meloxicam significantly inhibited the abilities of HepG2 cells to migrate, invade and adhere, by increasing E-cadherin expression and reducing MMP-2 expression. Addition of PGE2 almost completely abrogated these effects of meloxicam. In accord, PGE2 neutralized the inhibitory effect of NS-398, another COX-2 specific inhibitor, on MMP mRNA expression [4]. However, addition of rh-MMP-2 could only neutralize the effect of meloxicam on the expression of E-cadherin protein, but not mRNA. The results indicate that meloxicam regulates the expression of E-cadherin at post-translational levels through downregulating MMP-2, which has been shown to cleave full-length of E-cadherin into soluble E-cadherin [34].

The present study has shown that meloxicam inhibited the activation of the AKT pathway, and addition of PGE2 almost completely abrogated this inhibitory effect of meloxicam, indicating that meloxicam inhibits AKT activation in a COX-2-dependent way. It has been reported that overexpression of E-cadherin inhibits the PI3K/AKT pathway [35], [36], and soluble E-cadherin activates the PI3K/AKT pathway through human epidermal growth factor receptor (HER) and insulin-like growth factor-1 receptor (IGF-1R) [31] (Fig. 7). Thus, addition of rh-MMP-2 partly neutralized the inhibitory effect of meloxicam on AKT phosphorylation in the present study.

The results also showed that meloxicam induced apoptosis of HepG2 cells by downregulating the expression of Mcl-1 and survivin, and up-regulating the expression of Bax and Fas-L, indicating that meloxicam induces cell apoptosis via both intrinsic and extrinsic pathways. Addition of PGE2 almost completely abrogated the effects of meloxicam on the expression of survivin and Mcl-1, but failed to neutralize the effects of meloxicam on Bax and Fas-L, indicating that meloxicam induces cell apoptosis in both COX-2-dependent and -independent ways. In accord, meloxicam increased Bax expression majorly in a COX-2 independent pathway [37].

Autophagy is a cellular self-catabolic process in which cytoplasmic proteins and organelles are sequestered and delivered to lysosomes for degradation [38], and is defined as type II programmed cell death [39]. Autophagy is also a self-defense mechanism activated in response to stressful stimuli, such as anti-cancer drugs [40], [41], thus providing a mechanism for drug resistance in some cells [42]. Here, we have shown that meloxicam induced autophagy of HepG2 cells by upregulating expression of Beclin 1 and LC3. Both Beclin 1 [43] and LC3 [44] play central roles in cell autophagy. Specific inhibition of autophagy by 3-MA had little effect on cell apoptosis but could enhance the pro-apoptotic effects of meloxicam in HepG2 cells, accompanied by the further upregulation of Bax. However, the combination of meloxicam and 3-MA had little effect on the expression of Mcl-1, survivin and Fas-L. The antagonized effects of autophagy on meloxicam-induced apoptosis could be explained by that Bax may be partly engulfed by autophagosomes, supported by the previous reports that autophagy significantly attenuated the drug-induced apoptotic response [24], [42].

In summary, meloxicam executes its antitumor effects against HCC through multiple mechanisms in both COX-2-dependent and -independent pathways (Fig. 7). Firstly, meloxicam regulates apoptosis-associated proteins, survivin and Mcl-1, through activation of AKT by inhibiting the production of PGE2, as PGE2 effectively binds to its receptor, EP2, and in turn activates the AKT pathway. Secondly, meloxicam downregulates the expression of MMP-2 through inhibiting the production of PGE2; and downregulation of MMP-2 in turn increases the expression of E-cadherin, as MMP-2 cleaves full-length of E-cadherin [34]. Overexpression of E-cadherin inhibits [35], [36], but soluble E-cadherin activates, the PI3K/AKT pathway through HER/IGF-1R [31]. Thirdly, meloxicam-induced autophagy antagonizes its pro-apoptotic effect by reducing the expression of Bax as Bax may be engulfed by autophagosomes in the process of autophagy. Finally, meloxicam upregulates the expression of Bax and Fas-L in a COX-2-independent pathway. The results also indicate that inhibition of autophagy may enhance the therapeutic effects of meloxicam against HCC cells.

## Supporting Information

Figure S1 **Meloxicam inhibits the migration, invasion, adhesion and colony formation of SMMC-7721 cells.** (A) Representative photographs were taken from SMMC-7721 cells incubated for 48 h with meloxicam (80 μM) or vehicle (control) and subjected to cell migration, invasion, adhesion and colony formation assays as described in Materials and Methods. (B) The above assays were quantified. Data represent three independent experiments. “**” indicates a highly significant (*P*<0.001) difference from controls.
(TIF)

Figure S2 **Meloxicam induces apoptosis of SMMC-7721 cells.** SMMC-7721 cells were incubated for 72 h with meloxicam (80 μM) in the presence or absence of PGE2 (3 μM) or rh-MMP-2 (25 ng/mL). Untreated cells served as controls. (A) Representative dot plots were taken from cytometrically analyzed cells. (B) The apoptosis rate was calculated. Data represent three independent experiments. “*” indicates a significant (*P*<0.05) difference, and “**”, a highly significant (*P*<0.001) difference.
(TIF)

Figure S3 **Meloxicam induces autophagy of SMMC-7721 cells.** (A) Representative images were from SMMC-7721 cells that were incubated for 72 h with meloxicam (Mel) (80 μM) in the presence or absence of 3-MA (2 mM), and then stained by acridine orange. Untreated cells served as control (CTL). (B) The above cells from (A) were further subjected to flow cytometry to measure the degree of autophagic lysosomes as expressed by fold change of acridine orange fluorescence intensity (FL3) in treated cells versus control cells. Data represent three independent experiments. “**” indicates a highly significant (*P*<0.001) difference.
(TIF)

Figure S4 **Meloxicam induces autophagy of HepG2 and SMMC-7721 cells stained by MDC.** (A, C) Representative images were from HepG2 and SMMC-7721 cells that were incubated for 72 h with meloxicam (Mel) (80 μM) in the presence or absence of 3-MA (2 mM), and then stained by 0.05 mM MDC. Untreated cells served as control (CTL). (B, D) The above cells from (A, C) were further subjected to flow cytometry to measure the MDC-positive cells, respectively. Data represent three independent experiments. “**” indicates a highly significant (*P*<0.001) difference.
(TIF)

Figure S5 **Inhibition of autophagy by 3-MA promotes the apoptosis of SMMC-7721 cells.** SMMC-7721 cells were incubated for 72 h with meloxicam (80 μM) in the presence or absence of 3-MA (2 mM). (A) Representative dot plots were taken from cytometrically analyzed cells. (B) The apoptosis rate was calculated. Data represent three independent experiments. “**” indicates a highly significant (*P*<0.001) difference.
(TIF)

Figure S6 **Inhibition of autophagy by chloroquine (CQ) promotes apoptosis of HepG2 and SMMC-7721 cells.** HepG2 and SMMC-7721 cells were incubated for 72 h with meloxicam (80 μM) in the presence or absence of CQ (10 μM). (A, C) Representative dot plots were taken from cytometrically analyzed cells. (B, D) The apoptosis rate was calculated. Data represent three independent experiments. “**” indicates a highly significant (*P*<0.001) difference.
(TIF)
