# Supplementary figures and images for: Meloxicam Executes Its Antitumor Effects against Hepatocellular Carcinoma in COX-2- Dependent and -Independent Pathways
Source: PLoS One. 2014 Mar 27;9(3):e92864. doi: 10.1371/journal.pone.0092864 (PMC3968044; doi:10.1371/journal.pone.0092864)

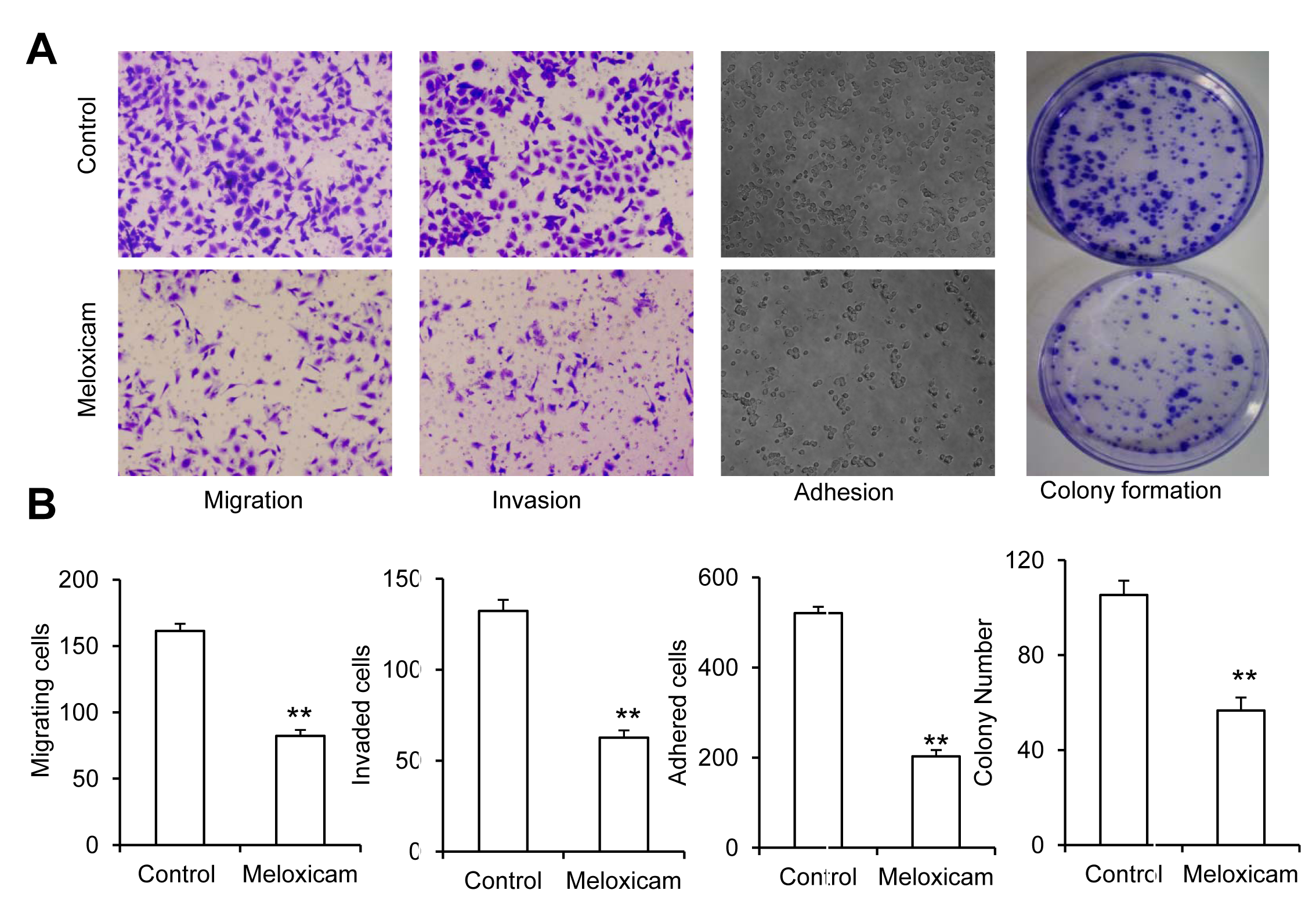

Supplement: Figure S1 — Meloxicam inhibits the migration, invasion, adhesion and colony formation of SMMC-7721 cells. (A) Representative photographs were taken from SMMC-7721 cells incubated for 48 h with meloxicam (80 μM) or vehicle (control) and subjected to cell migration, invasion, adhesion and colony formation assays as described in Materials and Methods. (B) The above assays were quantified. Data represent three independent experiments. “**” indicates a highly significant (P<0.001) difference from controls. (TIF) [file pone.0092864.s001.tif]

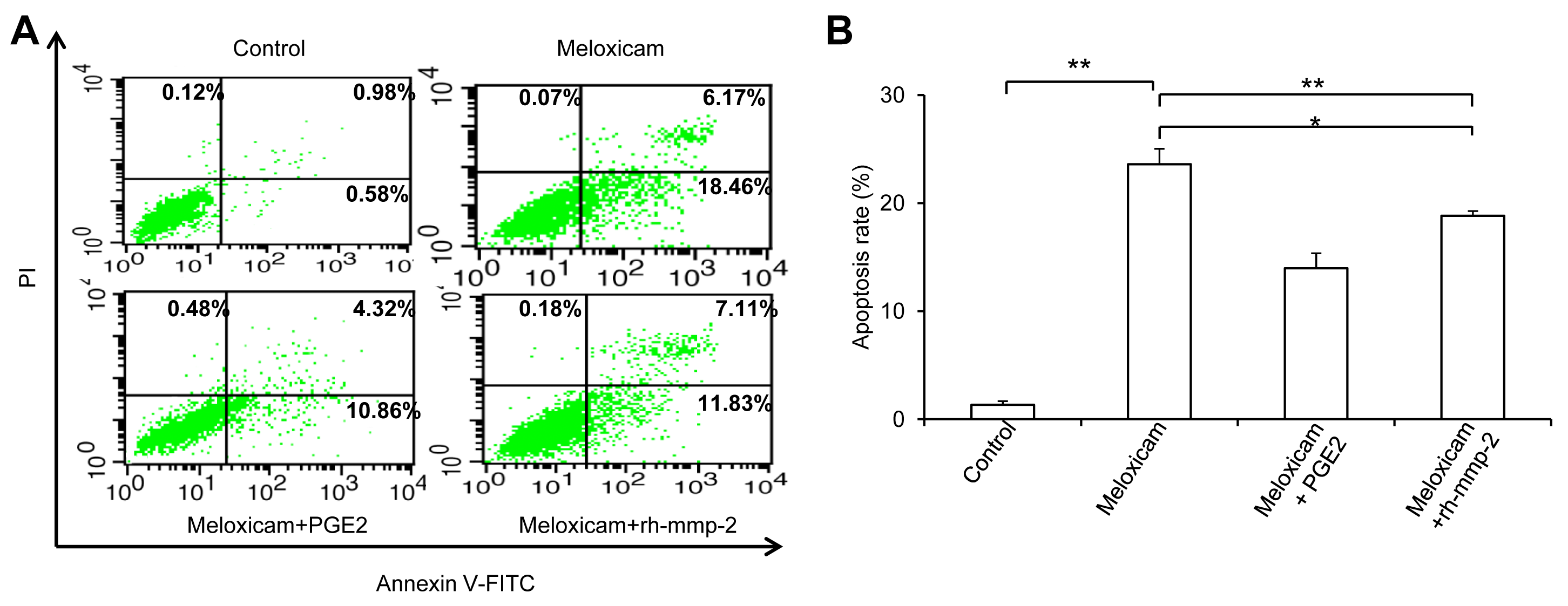

Supplement: Figure S2 — Meloxicam induces apoptosis of SMMC-7721 cells. SMMC-7721 cells were incubated for 72 h with meloxicam (80 μM) in the presence or absence of PGE2 (3 μM) or rh-MMP-2 (25 ng/mL). Untreated cells served as controls. (A) Representative dot plots were taken from cytometrically analyzed cells. (B) The apoptosis rate was calculated. Data represent three independent experiments. “*” indicates a significant (P<0.05) difference, and “**”, a highly significant (P<0.001) difference. (TIF) [file pone.0092864.s002.tif]

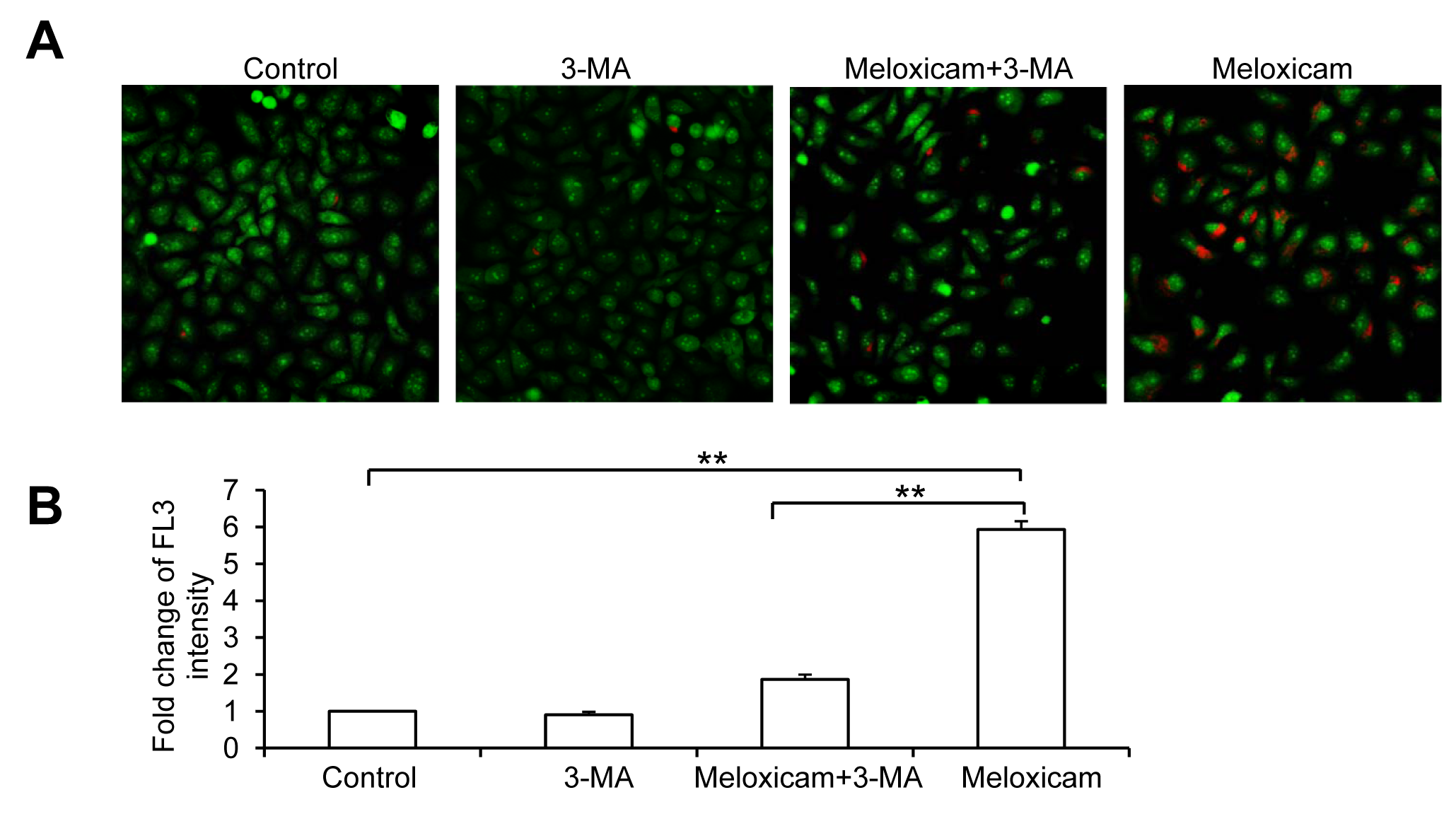

Supplement: Figure S3 — Meloxicam induces autophagy of SMMC-7721 cells. (A) Representative images were from SMMC-7721 cells that were incubated for 72 h with meloxicam (Mel) (80 μM) in the presence or absence of 3-MA (2 mM), and then stained by acridine orange. Untreated cells served as control (CTL). (B) The above cells from (A) were further subjected to flow cytometry to measure the degree of autophagic lysosomes as expressed by fold change of acridine orange fluorescence intensity (FL3) in treated cells versus control cells. Data represent three independent experiments. “**” indicates a highly significant (P<0.001) difference. (TIF) [file pone.0092864.s003.tif]

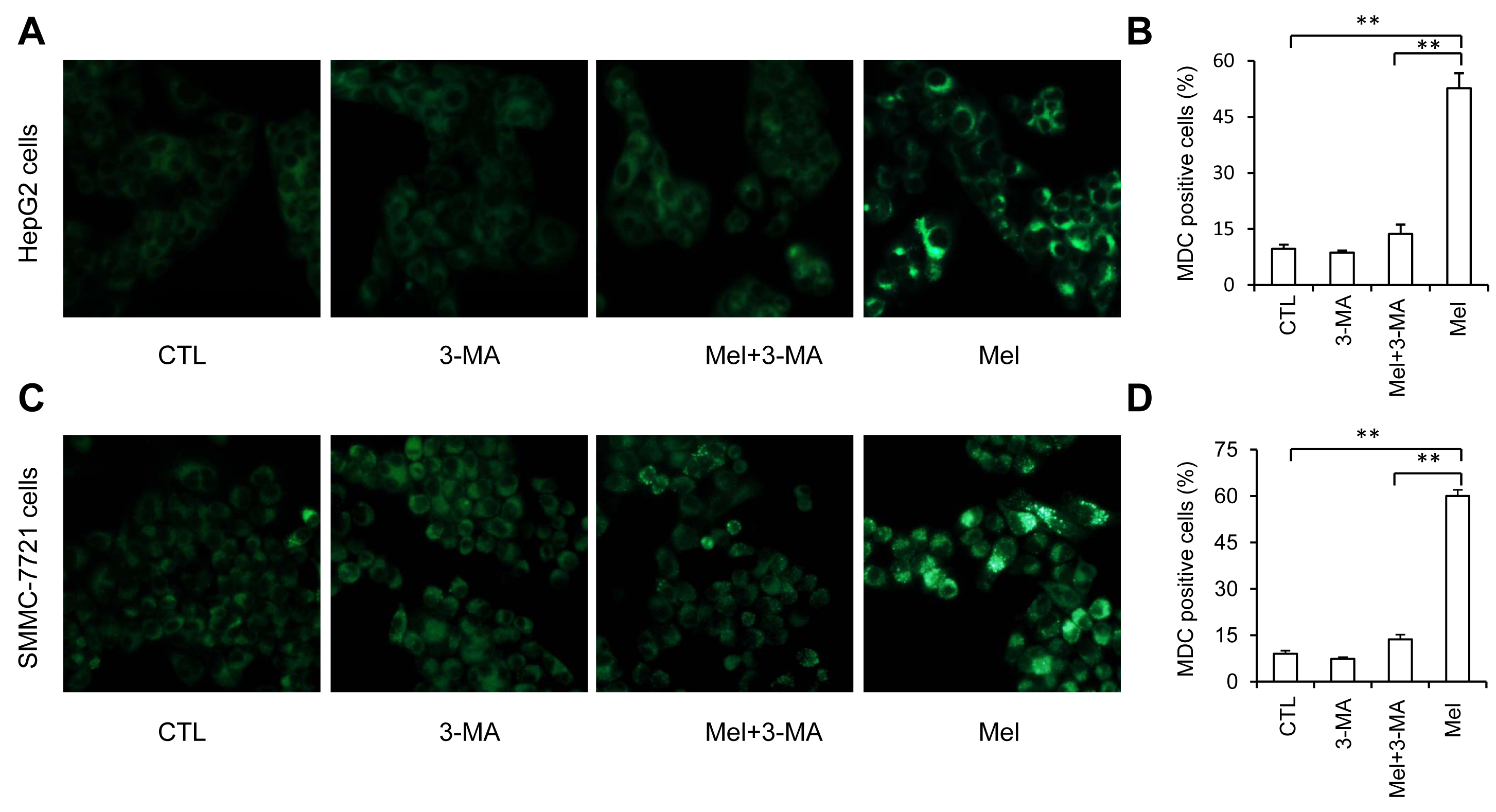

Supplement: Figure S4 — Meloxicam induces autophagy of HepG2 and SMMC-7721 cells stained by MDC. (A, C) Representative images were from HepG2 and SMMC-7721 cells that were incubated for 72 h with meloxicam (Mel) (80 μM) in the presence or absence of 3-MA (2 mM), and then stained by 0.05 mM MDC. Untreated cells served as control (CTL). (B, D) The above cells from (A, C) were further subjected to flow cytometry to measure the MDC-positive cells, respectively. Data represent three independent experiments. “**” indicates a highly significant (P<0.001) difference. (TIF) [file pone.0092864.s004.tif]

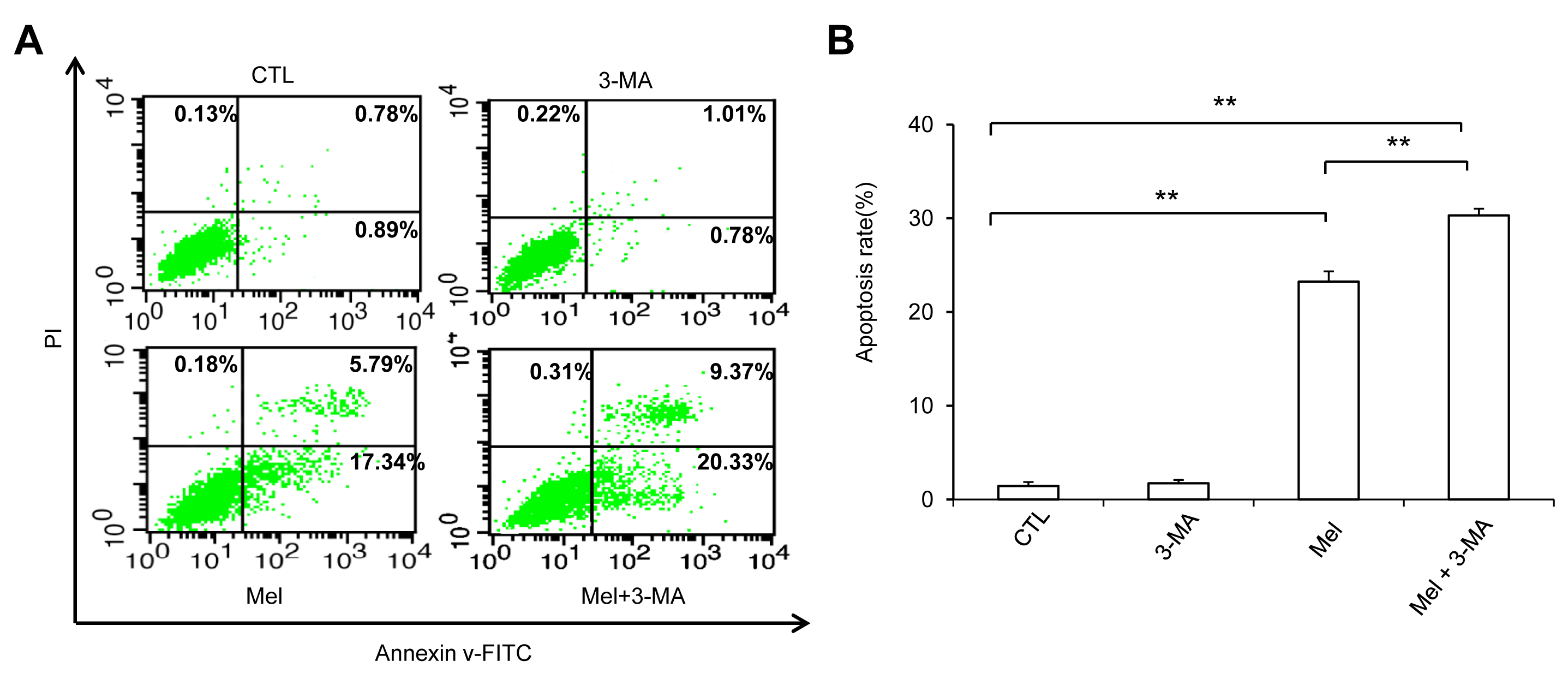

Supplement: Figure S5 — Inhibition of autophagy by 3-MA promotes the apoptosis of SMMC-7721 cells. SMMC-7721 cells were incubated for 72 h with meloxicam (80 μM) in the presence or absence of 3-MA (2 mM). (A) Representative dot plots were taken from cytometrically analyzed cells. (B) The apoptosis rate was calculated. Data represent three independent experiments. “**” indicates a highly significant (P<0.001) difference. (TIF) [file pone.0092864.s005.tif]

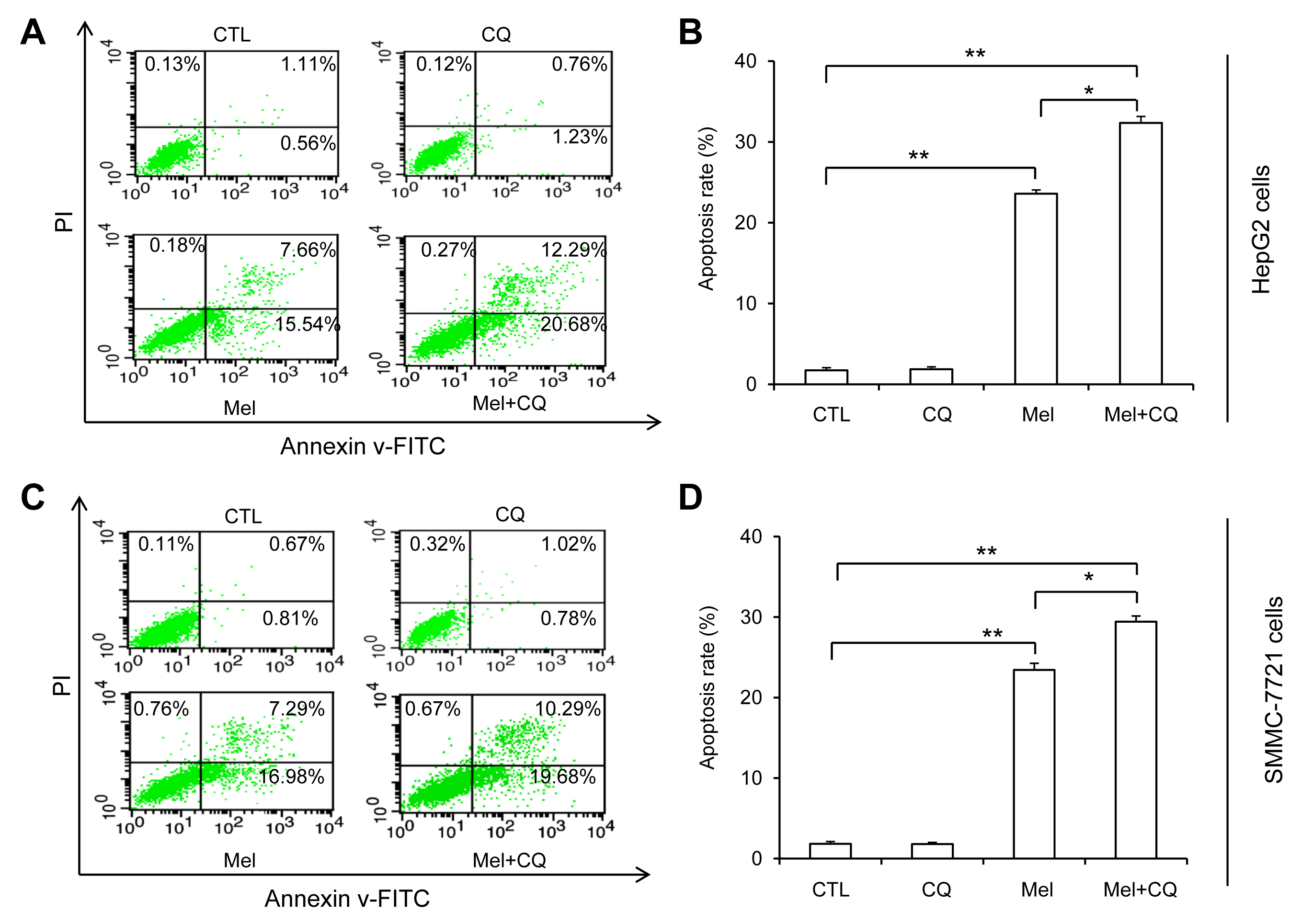

Supplement: Figure S6 — Inhibition of autophagy by chloroquine (CQ) promotes apoptosis of HepG2 and SMMC-7721 cells. HepG2 and SMMC-7721 cells were incubated for 72 h with meloxicam (80 μM) in the presence or absence of CQ (10 μM). (A, C) Representative dot plots were taken from cytometrically analyzed cells. (B, D) The apoptosis rate was calculated. Data represent three independent experiments. “**” indicates a highly significant (P<0.001) difference. (TIF) [file pone.0092864.s006.tif]
